# Combination vinblastine, prednisolone and toceranib phosphate for treatment of grade II and III mast cell tumours in dogs

**DOI:** 10.1002/vms3.106

**Published:** 2018-05-24

**Authors:** Jaime A. Olsen, Maurine Thomson, Kathleen O'Connell, Ken Wyatt

**Affiliations:** ^1^ Perth Veterinary Specialists Osborne Park Western Australia Australia; ^2^ Animal Referral Hospital Sinnamon Park Queensland Australia

**Keywords:** canine, chemotherapy, mast cell tumour, tyrosine kinase inhibitors, vinblastine

## Abstract

This retrospective study evaluates the progression‐free interval and survival outcomes of 40 canine (*Canis familiaris*) patients with Patnaik grade II and III mast cell tumours treated with combination vinblastine, prednisolone and toceranib phosphate from 2011 to 2015. Patients were subdivided into three groups; patients who received neoadjuvant therapy for poorly operable lesions, patients who received adjuvant therapy following surgical resection and patients being palliated for gross metastatic disease. Median survival time (MST) for the neoadjuvant group was not reached. Median survival time for the remaining groups was 893 days and 218 days, respectively. This combination demonstrated response in 90% (26/29) patients with measurable disease. The predominant side effects related to this chemotherapy combination were gastrointestinal in origin. Further prospective studies are required to further validate the efficacy of this treatment protocol.

## Introduction

Mast cell tumours (MCTs) are the most common cutaneous neoplasm of the dog, estimated to comprise 16–21% of these tumours. Mast cell tumours vary greatly in their gross appearance, biologic behaviour and malignancy (Blackwood *et al*. 2012). The aetiology of this disease remains poorly understood.

Treatment options in the published literature for canine MCTs are diverse. Options vary from surgical resection, radiation therapy, single or combination chemotherapy drug protocols or a combination of these (Gerritsen *et al*. 1998; Thamm *et al*. 1999; Séguin *et al*. 2001; Michels *et al*. 2002; Weisse *et al*. 2002; Dobson *et al*. 2004; Hahn *et al*. 2004; Thamm *et al*. 2006; Camps‐Palau *et al*. 2007; Hayes *et al*. 2007; Hahn *et al*. 2008; Rassnick *et al*. 2008; Cooper *et al*. 2009; London *et al*. 2009; Rungsipipat *et al*. 2009; Shida *et al*. 2009; Taylor *et al*. 2009; Hahn *et al*. 2010; Rassnick *et al*. 2010; Carlsten *et al*. 2012; Robat *et al*. 2012; Bernabe *et al*. 2013; Kry and Boston, 2014; Burton *et al*. 2015; Donnelly *et al*. 2015; Smrkovski *et al*. 2015). Due to the large number of treatment options described in the veterinary literature for this common neoplasm, many controversies still remain regarding a gold standard of management. There has been little consensus on the efficacy of each treatment modality between studies. This makes developing a gold standard of treatment difficult.

Receptor tyrosine kinase (RTK) inhibitors, including toceranib phosphate and masitinib mesylate, are drugs which have been recently developed and used for the management of MCTs in dogs. Receptor tyrosine kinases, including platelet‐derived growth factor receptor, vascular endothelial growth factor receptor and KIT receptors, are cell surface receptors. These are involved in the initiation of angiogenesis, promotion of cellular proliferation and metastasis in neoplastic cells such as in MCTs. Receptor tyrosine kinase inhibitor drugs act to competitively block the ATP‐binding sites on RTK receptors, preventing activation and downstream signalling (Blackwood *et al*. 2012; Robat *et al*. 2012).

Vinblastine is a plant alkaloid which binds tubulin, disrupting microtubule formation and therefore mitosis (Robat *et al*. 2012). Vinblastine has been frequently and successfully used, both as a single agent and in combination chemotherapy protocols, for the treatment of MCTs in dogs.

Reported response rates in gross MCT disease for single‐agent chemotherapy, including prednisolone, vinblastine, masitinib and toceranib, have varied from 27 to 75% (Dobson *et al*. 2004; London *et al*. 2009; Rungsipipat *et al*. 2009; Shida *et al*. 2009; Smrkovski *et al*. 2015). Response rates of 47–88% have been published for various chemotherapy combinations including prednisolone, CCNU and toceranib phosphate, with or without radiation therapy (Rassnick *et al*. 1999; Thamm *et al*. 1999; Dobson *et al*. 2004; Rassnick *et al*. 2008; Cooper *et al*. 2009; Rassnick *et al*. 2010; Carlsten *et al*. 2012; Robat *et al*. 2012; Burton *et al*. 2015).

To the best of the authors’ knowledge, there has only been one other published study regarding combination vinblastine and toceranib phosphate chemotherapy in canine patients with MCTs. This phase I study reported a 71% response rate, making this chemotherapy protocol an area of interest (Robat *et al*. 2012). The primary aim of this study is to describe the response of MCTs to a standard combination of vinblastine, prednisolone and toceranib phosphate in a clinical setting and determine survival times and progression‐free intervals for these patients. Identification of prognostic factors for these patients, as well as examination of available toxicity data were the secondary aims of this study.

## Materials and methods

### Study design

This study is a retrospective cohort study. It is based on the medical records from patients treated between 2011 and 2015 at Veterinary Oncology Specialists (VOS) at Veterinary Specialist Services (VSS), a referral hospital in Underwood, Queensland, Australia.

### Patient selection

Medical records from 40 client owned dogs with MCTs treated with combination vinblastine, prednisolone and toceranib phosphate (Palladia; Zoetis) were selected. All of the dogs included in the study had histologically confirmed MCTs of known Kiupel and Patnaik histologic grades, based on incisional or excisional biopsies. All patients in this cohort had Patnaik grade II and III MCTs, with both Kiupel grades represented. Patients were clinically staged and had histologic analysis of surgical margins if they underwent surgical excision. The pathologists used in this study varied. Clinical staging of these patients was based on the World Health Organization criteria from I to IV (Owen & World Health Organization, 1980). The staging protocol included full physical examination, complete blood count (CBC), serum biochemistry, thoracic radiographs, abdominal ultrasound and aspiration of lymph nodes or abdominal organs, if indicated based on imaging findings, for cytological analysis. Patients were excluded based on diagnoses made without histopathology and incomplete medical records. No exclusions were made based on clinical stage.

Patient characteristics, including signalment (age at diagnosis, breed, sex and spay or neuter status) and weight were recorded. Tumour characteristics recorded included location, grade, mitotic index, clinical stage, metastatic status at presentation and lesion size before and after chemotherapy administration (if measurable disease present). Results of a fortnightly CBC (done prior to each vinblastine dose) were also recorded.

The Veterinary Cooperative Oncology Group's (VCOG) response evaluation criteria for solid tumours in dogs was used to assess response to treatment (Nguyen *et al*. 2015). Partial response in these patients was defined as a physical reduction in tumour size of 30% of the largest diameter of the lesion or more. Complete remission was defined as resolution of disease with no remaining evidence of neoplasia following chemotherapy alone, based on either thorough physical examination or histopathology of tissue excised from the site of the original lesion (Nguyen *et al*. 2015). Additionally, completeness of surgical margins, response to treatment, if recurrence or metastatic spread occurred after initiation of treatment, complications resulting from treatment and if any other treatment protocols were used in this patient were noted. Local recurrence was defined as recurrence within 2 cm of the original MCT site. Progressive disease was defined as the appearance of one or more new lesions at a distant site to the primary lesion or a 20% or greater increase in the sum of diameters of the target lesions (Nguyen *et al*. 2015). Time, for the purposes of measuring survival outcomes, was measured in days from the date of first presentation at VOS for MCT disease.

### Subgroups

Patients were separated into three groups for the purpose of this study. Group 1 received four or more weeks of the vinblastine, toceranib phosphate and prednisolone protocol in a neoadjuvant setting (range 4–16 weeks). The purpose was to cytoreduce lesions in poorly operable locations, as determined by a boarded surgical oncologist, in order to make a lesion more amenable to surgical resection or to increase the likelihood of successful surgical removal. They then completed the remainder of the 16‐week protocol as adjuvant therapy.

Group 2 received a 16‐week course of this protocol as an adjuvant therapy following previous surgical resection. The majority of patients in this group had surgery performed at their referring veterinarian. These patients were selected for subsequent systemic therapy due to having a histological high‐grade MCT.

Group 3 received a median of 12 weeks of this chemotherapy protocol for management or palliation of gross metastatic disease. Patients in this group had gross lymph node involvement, with or without spread to abdominal organs, as determined through palpation and ultrasonography. Patients with progressive disease were offered alternate therapy and were censored at this time.

### Chemotherapy protocol

The chemotherapy protocol was consistent for patients in each subgroup. Chemotherapy was initiated an average of 7 days (range 0–55 days) following presentation. All medications were initiated concurrently on the same day and the majority were ceased simultaneously too, unless the animal was not in complete remission, as described below.

Vinblastine was administered at 1.6 mg m^−2^ IV every 2 weeks for a total of eight doses (16‐week course). Toceranib phosphate was dosed at 2.5 mg kg^−1^ orally on Monday, Wednesday and Friday for the duration of the 16‐week course. Prednisolone was administered at 1 mg kg^−1^ orally once daily for the duration of the course. Both prednisolone and toceranib phosphate were continued at the same dosing rates and intervals following completion of the protocol for up to 12 months unless the animal was in complete remission.

Patients selected for this study also received additional medications during their treatment for prophylactic management of paraneoplastic conditions associated with MCTs, as recommended in the European consensus document on mast cell tumours in dogs and cats (Blackwood *et al*. 2012). All patients received chlorpheniramine at 0.2–0.5 mg kg^−1^ orally every 12 h to reduce the impact of histamine on the peripheral vasculature and wound healing, and ranitidine 2–4 mg kg^−1^ orally every 12 h to treat and prevent gastric ulceration related to hyperhistaminaemia (Blackwood *et al*. 2012).

The protocol used for treating patients with toxicity depended on the duration of clinical signs. Animals presenting with diarrhoea had their dose of toceranib phosphate postponed until resolution of gastrointestinal signs. If the patient had ongoing diarrhoea or inappetence, they also received metronidazole and either metoclopramide or maropitant as an antiemetic. If the clinical signs persisted despite these additional medications, the dose of toceranib phosphate was reduced to 2.2 mg kg^−1^. If ongoing signs were present, the dosing frequency was reduced to twice weekly or the patient discontinued toceranib phosphate from their protocol, depending on the severity of ongoing toxicity.

### Statistical analysis

Statistical analysis was performed using the program ‘R’ (R Core Team, 2015).

Patients were separated into their three separate groups for statistical analysis. Survival data were censored if patients were lost to follow‐up, were alive at last follow‐up or were known to have died or be euthanized due to causes unrelated to their MCT. Patients whose cause of death was unknown were presumed to have died from MCT‐related complications. Patients were censored if their chemotherapy protocol was changed (excluding dose reductions) or if radiation therapy was introduced.

Outcome variables which were analysed were median survival time (MST) and progression‐free interval (PFI), measured in days. These were measured from the date of presentation. The risk variables which were assessed in this analysis were histologic grade (using both Patnaik and Kiupel grading systems), if disease was metastatic at presentation and response to chemotherapy (comparing partial and complete remission, as previously defined). The influence of these risk factors was assessed using a Cox proportional hazard model for both outcome variables.

Kaplan–Meier survival curves were plotted and MST and PFI were determined for the three subgroups of dogs. These were also used to compare individual risk variables’ effect on survival and progression‐free interval. A log‐rank test was used to compare survival curves. Multivariate analysis of risk factors was performed using a Cox proportional hazard model. A *P‐*value of < 0.05 was considered statistically significant for this study.

Any complications or toxic effects of chemotherapy drugs were recorded for each patient. The severity of these were classified using the VCOG common terminology criteria for adverse events version 1.1 (Veterinary Cooperative Oncology Group, 2016). Additionally, the outcomes and any treatments or management options, if required, were noted.

## Results

### Patient demographics

A total of 40 canine patients were selected from the VOS database based on the inclusion criteria listed. The median age of patients at presentation was 8 years and the median weight was 19 kg. Males and females were similarly represented in our cohort, with 18 female (15 spayed, three entire) and 22 male (18 neutered, four entire) patients above (see Table 1 for patient characteristics). Staffordshire Bull Terriers were the most common breed in our cohort, making up 30% (12/40) of the patients. Other breeds represented included mixed breed dogs, Shar Peis and Boxers.

**Table 1:** Patient demographics at presentation

Characteristics	Value	Range
Total participants	40	
Median age at presentation (years)	8	3–13
Median weight (kg)	19	4–59
Gender		
Female spayed	15	
Female intact	3	
Male castrated	18	
Male intact	4	
Breed		
Staffordshire Bull Terrier	12	
Mixed breeds	6	
Shar Pei	4	
Boxer	3	
Pug	2	
Other	13	
Tumour Grade (Patnaik/Kiupel)		
Grade III/High	10	
Grade II/High	11	
Grade II/Low	19	
Metastatic disease at presentation		
Local lymph node	16	
Distant and visceral	8	
Median Mitotic Index (MI/10 hpf)^a^	2	0–72

a Four patients did not have MI data available.

Patients predominantly had Patnaik grade II MCTs (*n* = 29), consisting of both Kiupel low (*n* = 19) and high (*n* = 10) grades. The remaining patients (*n* = 11) had Patnaik grade III, Kiupel high‐grade MCTs. The median mitotic index was 2 MI/10 hpf (range 0–72 MI/10 hpf). Sixty per cent (24/40) of patients had metastatic spread at presentation. Of these patients, 66% (*n* = 16) had local lymph node involvement and 33% (*n* = 8) had distant or visceral metastatic disease. Overall, patients in this study had an MST of 893 days (range 13–1290 days) and a PFI of 317 days (range 0–1229 days).

### Group 1 – Neoadjuvant chemotherapy

This group consisted of patients (*n* = 16) which received vinblastine and toceranib phosphate for chemotherapeutic downstaging of their MCTs in a neoadjuvant setting. This was an attempt to better facilitate surgical resection or to improve surgical margins when the site of the lesion was considered poorly operable by a single boarded surgical oncologist. Locations of these tumours included the nasal planum, vulva, prepuce, anus and retrobulbar region.

The majority of dogs in this group had Patnaik grade II MCTs (consisting of both Kiupel low grade (*n* = 13) and high grade (*n* = 1)), with two dogs having Patnaik grade III MCTs. The median mitotic index in these tumours was 1 MI/10 hpf (range 0–20 MI/10 hpf). The clinical stages of patients in this group ranged from I to IV. Metastatic disease was present in 50% (8/16) patients at presentation, consisting of six patients with local lymph node involvement and two patients with distant metastases. Seven of the patients presenting with metastatic disease had Patnaik grade II disease, with the remaining patient having grade III disease. Four patients were censored from MST and PFI statistical analysis at the time that alternate therapies were initiated, due to disease progression.

The patients in this group which underwent surgical resection (*n* = 12) received a median of 6 weeks of the chemotherapy protocol pre‐operatively (range 4–16 weeks), then continuing the protocol post‐operatively. The remaining four dogs, which did not undergo surgical excision, completed 16 weeks of the protocol. All patients which underwent surgery and had local lymph node metastases (*n* = 5) also had the metastatic lymph node removed at the time of surgery. The primary tumours in these dogs were measured throughout treatment in order to gauge response to chemotherapy. Eighty‐eight per cent (14/16) of animals in this group had a measurable response to chemotherapy, including both complete and partial responses. A complete response to medical downstaging alone occurred in 38% (6/16) patients. Four of these dogs had complete gross resolution of their primary lesions with medical therapy only; therefore, plans for surgical excision did not proceed. The other two dogs underwent surgery due to the lesion remaining grossly evident but had no histologic evidence of neoplasia throughout the resected tissues. Of the patients which went to surgery with histologic evidence of neoplasia, complete surgical margins were achieved in 70% (7/10).

Local disease recurrence occurred in two dogs at 112 and 160 days. These patients had incomplete surgical margins. One of these patients underwent radiation therapy following chemotherapy, with the other dog not receiving any further local treatment. Two of the six animals (33%) which had a complete response to therapy went on to have de novo lesions at follow‐up. There was no statistical significance between MST (*P* = 0.683) or PFI (*P *=* *0.981) for individuals who had complete and incomplete surgical margins.

**Figure 1:**
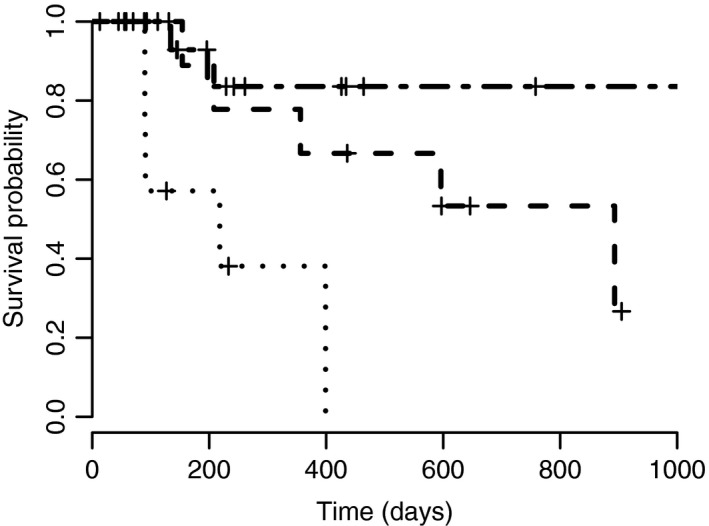
Kaplan–Meier curve for survival time. Group 1 (neoadjuvant chemotherapy) is depicted using a dot‐dash line, group 2 (adjuvant chemotherapy) is depicted using a dashed line and group 3 (palliative chemotherapy) is depicted using a dotted line. Time of censorship of a patient is depicted with a cross.

**Figure 2:**
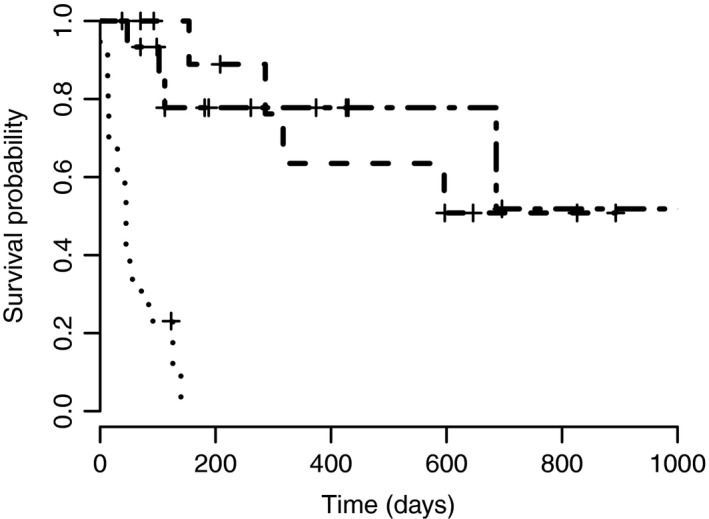
Kaplan–Meier curve for progression free interval. Group 1 (neoadjuvant chemotherapy) is depicted using a dot‐dash line, group 2 (adjuvant chemotherapy) is depicted using a dashed line and group 3 (palliative chemotherapy) is depicted using a dotted line. Time of censorship of a patient is depicted with a cross.

Median survival time and PFI for patients which underwent neoadjuvant chemotherapy were not reached with a median follow‐up time of 287 days (range 84–1290 days). Twenty‐five per cent (4/16) of dogs died from causes attributed to MCT disease. Of these patients, three dogs had Patnaik grade II lesions (consisting of Kiupel low grade (*n* = 2) and high grade (*n* = 1)) and one dog had Patnaik grade III disease. See Figs 1 and 2 for Kaplan–Meier survival curves.

### Group 2 – Adjuvant chemotherapy

The second subgroup of dogs (*n* = 11) were administered chemotherapy in an adjuvant setting. Patients in this group had Patnaik grade II (*n* = 7) and III MCTs (*n* = 6), with all these lesions being classified as Kiupel high grade. The median MI of the MCTs in these patients was 10 MI/10 hpf (range 2–72 MI/10 hpf). The majority of surgical procedures were performed by their referring veterinarian prior to referral, meaning the surgeon and surgical skill was likely variable among this group. Margins were all histologically assessed following resection. Clean surgical margins were achieved in 82% (9/11) of cases, with the remaining two lesions demonstrating neoplastic mast cells extending into the margins. These patients received a median of 16 weeks (eight vinblastine doses) of the protocol (range 8–26 weeks) as part of their treatment. Two patients received a longer protocol (26 weeks and 22 weeks) due to progressive disease.

Metastatic disease was present in 27% (3/11) of dogs at presentation, located in the regional lymph node. Two of these patients underwent removal of the affected node following referral, with the remaining patient demonstrating adequate reduction in lymph node size following chemotherapy that surgery was not deemed necessary.

Disease progression occurred in 36% (4/11) of dogs, with locally recurrent disease occurring in two animals and metastatic spread in the other two dogs. Both animals with local recurrence had incomplete surgical margins and did not undergo any further local therapy, such as re‐excision or radiation. Median survival times for complete and incomplete surgical margins were 893 days (range 70–893 days) and 181 days (range 70–548 days), respectively. Progression‐free interval was not reached for complete surgical margins and incomplete surgical margins had a PFI of 154 days (range 70–597 days). A statistically significant difference in survival times (*P *=* *0.00186) and PFI (*P *=* *0.00323) was shown between animals with complete compared to incomplete margins on univariate analysis. Median survival time for Patnaik grades II and III were 208 days (range 70–646 days) and 893 days (range 93–905 days), respectively. No statistically significant difference was seen when comparing Patnaik grades (*P *=* *0.197). At last follow‐up, 45% (5/11) of the animals were alive and remained in complete remission. The remaining six dogs were deceased, all attributed to MCT disease. The MST of this group was 893 days (range 70–905 days). The median PFI was not reached over a median follow‐up time for animals in remission of 597 days (70–905 days). See Figs 1 and 2 for Kaplan–Meier survival curves.

### Group 3 – Gross metastatic disease

The third subgroup consisted of dogs (*n* = 13) which were to receive a 16‐week course of vinblastine, toceranib and prednisolone chemotherapy, for management of, or palliation of gross metastatic disease. The length of treatment was largely dictated by patients exhibiting progressive disease, necessitating a change of protocol. The patients in this subgroup received a median of 12 weeks (six vinblastine doses) of the protocol. If patients went into remission following their vinblastine doses, they continued on prednisolone and toceranib therapy only for up to 12 months, in an aim to minimize progressive disease. Seven individuals had histological high‐grade disease determined using the Kiupel system; two of these were grade III and five were grade II using the Patnaik system. The remaining six individuals had Patnaik grade II, Kiupel low‐grade disease. Mitotic index was available for 10 patients’ tumours. The median mitotic index was 3 MI/10 hpf (range 0–30 MI/10 hpf). The clinical stage of these animals on presentation ranged from II to IV. Three of the patients in this group had undergone local treatment on the primary lesion, including surgery, prior to starting this protocol and had inadequate local control of their lesions.

All the patients in this group had gross metastatic spread of their mast cell neoplasia, as determined by palpation and confirmed using cytology. Local lymph node involvement only, without further spread, was seen in 54% (7/13) of dogs. Involvement of multiple distant lymph nodes with no visceral spread occurred in 23% (3/13) and cytology confirmed involvement of abdominal or thoracic organs in addition to lymph node involvement in the remaining 23% (3/13) of dogs.

A response occurred in 92% (12/13) patients in this group following chemotherapy, including both partial and complete responses. A complete response was seen in 23% of dogs (*n* = 3) and a partial response in 69% (*n* = 9). All patients with a complete response had grade II MCTs, with two displaying high‐grade disease and the remaining patient having Kiupel low‐grade disease. The clinical stages of these patients ranged from II to III.

Other rescue treatment protocols, including intralesional therapy, radiation and alternative systemic chemotherapy agents, were administered to 54% (7/13) of patients in this group following treatment with the vinblastine, prednisolone and toceranib phosphate protocol due to progressive disease. These animals were censored at the time of initiating a new protocol for the purposes of MST and PFI statistical analysis.

The MST for all patients in this group was 218 days (range 90–399 days). MSTs for individuals with local lymph node involvement only (*n* = 7), multiple distant lymph node involvement (*n* = 3) and visceral metastases (*n* = 3) were 218 days (range 90–233 days), 399 days (range 45–399 days) and 90.5 days (90–91 days), respectively.

The PFI for these patients was 45 days (range 0–140 days). Progression‐free intervals for individuals with local lymph node involvement only (*n* = 7), multiple distant lymph node involvement (*n* = 3) and visceral metastases (*n* = 3) were 43 days (range 13–140 days), 84 days (range 45–126 days) and 14 days (0–56 days), respectively. See Figs 1 and 2 for Kaplan–Meier survival curves. There was no statistically significant difference in survival times or PFI between dogs when comparing local lymph node involvement and visceral disease (survival *P *=* *0.256 and PFI *P *=* *0.338), local lymph node involvement and distant lymph node involvement (survival *P *=* *0.281 and PFI *P *=* *0.745) and distal lymph node involvement and visceral disease (survival *P *=* *0.0896 and PFI *P *=* *0.11).

Progression of MCT disease occurred in 92% (12/13) of these patients. These animals all died or were euthanized due to progressive disease. The remaining patient died of unrelated seizures leading to cardio‐respiratory arrest, without evidence of progressive MCT disease.

### Prognostic factors

Factors investigated using the Cox Proportional Hazard Model were tumour grade (both Patnaik and Kiupel), if disease was metastatic at presentation and whether the animal had a partial or complete response to chemotherapy. Analysis of this data did not identify any statistically significant prognostic factors in our cohort for survival time or PFI (see Table 2).

**Table 2:** Cox proportional hazard analyses for clinical outcomes for 40 dogs with mast cell tumours

Risk factor	Hazard ratio	95% Confidence interval	*P‐*value
Survival time			
Histologic grade (Kiupel)^a^	3.252	0.73–14.49	0.12
Histologic grade (Patnaik)^b^	0.374	0.07–2.01	0.25
Response to chemotherapy^c^	0.625	0.15–2.63	0.52
Metastatic disease^d^	1.462	0.43–5.03	0.55
Progression free interval			
Histologic grade (Kiupel)^a^	1.87	0.73–14.49	0.29
Histologic grade (Patnaik)^b^	0.753	0.07–2.01	0.676
Response to chemotherapy^c^	0.514	0.15–2.63	0.259
Metastatic disease^d^	2.68	0.43–5.03	0.054

a Histologic grade using Kiupel grading scheme – consisting of high and low grades.

b Histologic grade using Patnaik grading scheme – consisting of grade 1, grade 2 and grade 3.

c Response to chemotherapy – consisting of partial and complete response.

d Metastatic disease present at first presentation.

### Toxicity

Toxicity was noted in 67% (27/40) patients who received this chemotherapy protocol. The majority of toxic effects were gastrointestinal and musculoskeletal (weakness or lameness), seen in 81% (22/27) and 48% (13/27) of patients experiencing side effects, respectively. Other side effects in this cohort included lethargy, inappetence, myelosuppression (anaemia, neutropenia and thrombocytopenia), epistaxis, dermatological disease, polyuria and polydipsia, increased liver enzymes (including alkaline phosphatase (ALP) and alanine transaminase (ALT)), azotaemia and weight loss (see Table 3). Neutropenia, the dose‐limiting toxicity for vinblastine, was seen in four patients. Three of these patients were classed at grade I and the remaining patient was grade III (grade I is defined as 1500 *μ*L^−1^‐LLN, grade II as 1000–1499 *μ*L^−1^, grade III as 500–999 *μ*L^−1^ and grade IV as <500 *μ*L^−1^) (Veterinary Cooperative Oncology Group, 2016).

**Table 3:** Adverse effects and treatment required

Types of side effects	Number of dogs affected				
	Grade 1	Grade 2	Grade 3	Grade 4	Total
Gastrointestinal (vomiting or diarrhoea)	18	2	2	0	22
Musculoskeletal (weakness or lameness)	7	4	2	0	13
Lethargy or inappetence	3	1	2	0	6
Anaemia	2	1	1	2	6
Neutropenia	3	0	1	0	4
Dermatological	2	1	0	0	3
Epistaxis	2	0	0	0	2
Thrombocytopenia	1	0	0	1	2
Polyuria and polydipsia	2	0	0	0	2
Increased liver enzymes (ALP/ALT)	0	0	2	0	2
Weight loss	0	1	1	0	2
Azotaemia	1	0	0	0	1
Treatment			Number of dogs treated		
Temporary discontinuation/lowered dose of Toceranib			12		
No treatment			7		
Complete discontinuation of therapy			4		
Hospitalization for medical management			2		
Euthanasia			1		
Chemotherapy discontinuation due to unrelated disease			1		

The management required in affected patients were most commonly temporary discontinuation or lowered dosage of toceranib phosphate in 44% of dogs (*n* = 12/27) or no treatment in 26% (*n* = 7/27). Fifteen per cent (*n* = 4/27) of patients required complete discontinuation of therapy and two animals required hospitalization for medical management and supportive care to treat their condition.

## Discussion

This aims of this study were to examine the efficacy and safety profile of a relatively novel chemotherapy combination. A single phase I trial of this drug combination has shown promise in its efficacy and safety for treating mast cell tumours (Robat *et al*. 2012). Additionally, other protocols involving RTK inhibitors have proven these to be valuable drugs in the management of MCT disease (London *et al*. 2009; Carlsten *et al*. 2012; Burton *et al*. 2015; Smrkovski *et al*. 2015). This study aims to expand on already established knowledge of toceranib phosphate, vinblastine and prednisolone combination chemotherapy and further establish the efficacy of this combination in the treatment of canine MCTs in various clinical settings, particularly those considered high risk for development of metastatic disease.

The dose rates of drugs which have been used in this study protocol have been reduced compared with recommended doses for single‐agent chemotherapy. The dose of toceranib phosphate used in this study was 2.5 mg kg^−1^ three times a week, compared with the recommended single‐agent dosage of 3.25 mg kg^−1^ EOD (Yancey *et al*. 2010). Lowered dose rates of toceranib phosphate have been associated with less adverse effects when combined with vinblastine, while still maintaining therapeutic levels suitable for target inhibition. Despite this, the label dose may still be used relatively safely for combination chemotherapy protocols; however, the risk of dose limiting myelosuppression and gastrointestinal toxicity is greater (Robat *et al*. 2012; Bernabe *et al*. 2013). Additionally, the dose of vinblastine used in this protocol was 1.6 mg m^−2^, which is also significantly reduced from the recommended dosage for single‐agent use. Effective and tolerable dose rates for vinblastine in patients with MCTs have been reported as 2–3.5 mg m^−2^ (Rassnick *et al*. 2008; Plumb, 2011; Serra Varela *et al*. 2016). The dosage used in this study was extrapolated from the safety study by Robat *et al*. (2012). The efficacy of this protocol, despite lowered dose rates, indicates there may be synergism between drugs in this protocol. It should be noted though, that it is difficult to identify which of the drugs are effective when administering a multi‐drug protocol.

In terms of other medications administered to these patients for management and prevention of paraneoplastic conditions, the use of ranitidine as a gastric acid suppressant has been brought into question. Literature on this topic suggests that other medications, including omeprazole, are more efficacious in canine patients for gastric acid suppression, with ranitidine shown to be poorly efficacious (Bersenas *et al*. 2005). An alteration to this portion of the drug protocol should be considered for patients undergoing this chemotherapeutic treatment and for use in future studies.

Clinical staging was performed on all patients in this study. A potential limitation of the methods implemented for staging patients is the predominant use of diagnostic imaging techniques for detection of abdominal metastatic disease. Cytology was only performed in the presence of ultrasonographic abnormalities. There is significant controversy regarding the relevance of abnormalities on imaging and correlation with cytology. One study demonstrated that ultrasound as a stand‐alone diagnostic technique for detection of abdominal MCT metastases has a poor sensitivity, ranging from 0 to 43%, depending on the organ assessed. Uniform cytology of these organs was recommended for detection of metastatic disease (Book *et al*. 2011). Similarly, there is evidence that, when determining the presence of nodal metastases, uniform cytology in all patients with MCT had greater sensitivity, and was appropriately correlated with grade and outcome (Krick *et al*. 2009; Mutz *et al*. 2017). Conversely, another study has discredited the clinical relevance of uniform cytological screening of patients with MCT disease, if no liver or splenic abnormalities were detected using ultrasound. This study found that the presence of mast cells in routine cytology of the liver and spleen was not a reliable indicator of malignancy (Finora *et al*. 2006). These studies suggest that the staging techniques used in this study may be a limiting factor, and may have led to underestimation or overestimation of the clinical stages of these patients.

Surgical resection of the primary lesion with wide margins has long been regarded an integral component in the treatment of MCTs in canine patients (Séguin *et al*. 2001; Michels *et al*. 2002; Weisse *et al*. 2002; Murphy *et al*. 2004; Donnelly *et al*. 2015). Group 1 included patients with MCTs which underwent neoadjuvant cytoreduction, in an aim to improve the likelihood of successful surgical excision and therefore local control of the primary tumour. The inclusion of many of the cases in group 1 was based on poor candidacy for effective surgical resection on presentation rather than pre‐treatment grade or clinical stage. This technique was used to facilitate surgery in tumours in locations in which surgical resection would otherwise lead to poor cosmesis, functional compromise or limb amputation. Considering that local control is generally deemed a major component in the management of MCT disease, it is reasonable to believe that facilitating surgical excision or radiation therapy where local control would otherwise be unachievable is likely to improve outcomes for these patients and may reduce surgical morbidity in some locations.

The patients in group 1 responded well to the protocol in question, as evidenced by an 88% response rate and a subjective improvement in the amenability of the tumour to surgery, as determined by a boarded surgical oncologist. Response rates to other protocols in patients with gross measurable disease, involving single‐agent and combination therapies composed of toceranib, vinblastine, CCNU, prednisolone, cyclophosphamide and chlorambucil ranged from 38 to 71% (Rassnick *et al*. 1999; Thamm *et al*. 1999; Camps‐Palau *et al*. 2007; Stanclift and Gilson, 2008; Cooper *et al*. 2009; London *et al*. 2009; Taylor *et al*. 2009; Robat *et al*. 2012; Burton *et al*. 2015). It is important to note that prednisolone, as a single‐agent therapy, resulted in an overall response rate of 70% (Stanclift and Gilson, 2008). This protocol is both inexpensive and has less severe adverse effects than combination therapies, such as the one in question, and should be compared with multi‐drug protocols in future studies. However, it should also be considered that prednisolone alone does not carry a favourable MST, with one study demonstrating an MST of 175 days (Rungsipipat *et al*. 2009).

The MST and PFI for group 1 were not reached over a median follow‐up time of 287 days. Other studies looking at neoadjuvant therapy for inoperable lesions or treatment of gross disease have demonstrated varied results for patient survival. Chemotherapy protocols, consisting of a single agent or combinations of prednisolone, vinblastine, CCNU, cyclophosphamide, chlorambucil and toceranib, had MSTs ranging from 53 days to 35 weeks (Rassnick *et al*. 1999; Camps‐Palau *et al*. 2007; Cooper *et al*. 2009; Taylor *et al*. 2009; Burton *et al*. 2015). CCNU and combination prednisolone and vinblastine had PFIs of 77 days and 154 days, respectively (Rassnick *et al*. 1999; Thamm *et al*. 1999). MCTs treated various combinations of prednisolone, toceranib and radiotherapy had PFIs of 316–1031 days (Dobson *et al*. 2004; Carlsten *et al*. 2012). In comparison, the results from this study indicate relatively good survival times, but with some therapies demonstrating better effects. However, studies in a larger study population are warranted to improve knowledge of this chemotherapy combination.

Clinical staging of patients in group 1 ranged from I to IV, as determined prior to initiation of therapy. Two patients were clinically staged as stage IV, involving distant metastatic disease. These patients underwent neoadjuvant therapy in an aim to facilitate control of their primary disease, and secondarily manage the metastatic disease. In patients with metastatic MCT disease, surgical resection of the primary tumour in combination with adjuvant chemotherapy has been demonstrated to confer a significant survival advantage over surgical management alone (Miller *et al*. 2016). Another study has demonstrated that achieving adequate local tumour control in animals with stage IV disease is correlated with a better outcome (Pizzoni *et al*. 2018). This further reinforces the importance of focussing on local control, despite the presence of distant metastases. Despite the high clinical stage of the patients in this study, both dogs had good outcomes, and remained in complete remission at last follow‐up. For future studies, inclusion of patients with higher clinical stages in a neoadjuvant chemotherapy protocol may be inappropriate, due to the secondary disease present.

Even though the majority of the lesions in subgroup 1 were Kiupel low grade, the metastatic rate was 50%, which was higher than would be expected, categorizing these patients’ disease ‘high‐risk’. This may have been due to many of these tumours being mucocutaneous, or at sites often considered to have a higher biological behaviour than their pathology indicates, including the head, muzzle, oral and perioral lesions (Gieger *et al*. 2003; Dobson *et al*. 2004; Hillman *et al*. 2010; Elliott *et al*. 2016). It should be noted that the predominance of low grade lesion in this subgroup may have produced a survival advantage.

The authors recognize the risk for neoadjuvant therapy to grossly reduce tumour volume, while allowing progression of lateral and deep microscopic margins. Despite the tumours in patient in group 1 being initially considered poorly operable or inoperable, the majority were able to be surgically resected with histopathological clean margins following downstaging with chemotherapy. Additionally, six dogs went into complete remission, with two of these lesions having no evidence of neoplastic cells on histopathology. This information suggests that both gross and microscopic size reduction in mast cell tumours may be an achievable goal with many of these tumours. This provides options to practitioners for improving surgical outcomes in patients with tumours in poorly operable sites and for improving local control when performing excisional surgery.

A benefit for adjuvant chemotherapy following complete resection of grade II MCTs has been debated in the literature. There have been studies demonstrating that complete surgical excision alone is appropriate in most patients, with a relatively low rate of local recurrence reported of 5–11%. This makes further local or systemic adjuvant treatment unnecessary (Séguin *et al*. 2001; Weisse *et al*. 2002). Conversely, other studies have advocated that adjuvant therapy, in cases of MCTs which are high risk for metastatic disease, can improve survival, compared with surgery alone (Thamm *et al*. 2006; Miller *et al*. 2016). The significance of surgical margins in Patnaik grade II or Kiupel low‐grade MCTs should be considered when determining if adjuvant therapy is appropriate. Some studies suggest minimal to no prognostic difference between patients with complete and incomplete histological margins (Michels *et al*. 2002; Murphy *et al*. 2004; Donnelly *et al*. 2015). Others have shown more effective control when complete surgical margins are achieved (Séguin *et al*. 2001; Weisse *et al*. 2002). Incomplete margins may therefore be an indicator that further local therapy, including surgical re‐excision and radiation therapy, or systemic therapy should be implemented. Adjuvant therapy (both chemotherapeutic or radiation therapy) is generally recommended in the treatment regime for grade III or high‐grade MCTs, regardless of margins. These patients are at high risk of local recurrence and metastatic disease. They have been shown to have relatively short survival times if surgical treatment alone is used and have an overall poorer prognosis for survival (Thamm *et al*. 1999; Murphy *et al*. 2004; Thamm *et al*. 2006; Hayes *et al*. 2007; Rassnick *et al*. 2010; Blackwood *et al*. 2012; Donnelly *et al*. 2015). For these reasons, patients in group 2, with Kiupel high‐grade lesions, were selected to have adjuvant chemotherapy following surgery.

The patients in group 2, having undergone adjuvant chemotherapy, had an MST of 893 days. The PFI for this group was 597 days. Comparatively, the MST for cases of grade III MCTs treated with surgery alone was 278 days (Murphy *et al*. 2004). Reported MSTs for patients receiving adjuvant chemotherapy, including single‐agent and combinations of prednisolone, vinblastine, CCNU and masitinib, ranged from 175 days to 1946 days. The reported MST for patients receiving adjuvant prednisolone and vinblastine ranged from 322 days to 1946 days (Thamm *et al*. 2006; Hayes *et al*. 2007; Miller *et al*. 2016). Prednisolone as a stand‐alone therapy has an MST of 175 days in grade III MCTs (Rungsipipat *et al*. 2009). Patients treated with adjuvant prednisolone, vinblastine and lomustine in two studies had a MST and PFI of 1103 days and 489 days, respectively (Rassnick *et al*. 2010; Lejeune *et al*. 2015). Adjuvant masitinib had a MST of 396 days (Miller *et al*. 2016). The survival data for toceranib, prednisolone and vinblastine chemotherapy was favourable, considering the high‐risk nature of the MCT disease in these patients. Additionally, there was a statistically significant survival advantage for patients with complete surgical margins, compared to those with incomplete margins. This further reinforces the importance of local disease control. This also supports the use of adjuvant therapies in high‐risk cases or those in which incomplete surgical margins were achieved, as it may confer advantages for patient outcome when compared with stand‐alone surgical management. However, further research into this area is required to further refine protocols for adjuvant treatment in veterinary patients, as the results of these methods vary greatly throughout the literature.

Three patients in group 2 had metastatic disease to the regional lymph node on presentation, following resection of the primary lesion by the referring veterinarian. Two of these patients underwent subsequent surgical intervention for removal of the lymph nodes of concern. One patient did not undergo removal of the affected nodes, due to adequate gross reduction in lymph node size. It should be noted however, that this could potentially pose a risk to the patient, as the node could still harbour neoplastic cells, despite gross reduction in size.

In relation to the use of this chemotherapeutic combination for palliation of gross metastatic disease, it is difficult to assess the merit of this treatment. Sample size for this group was small and the period in which they received this treatment protocol without alternative rescue therapies was relatively short. For this reason, no statistically significant conclusions could be made. Despite a lack of statistical significance, patients in this group with visceral metastatic disease had a shorter PFI and MST in comparison to individuals with local and distant lymph node involvement. The possibility of visceral metastatic disease being a negative prognostic indicator for both progressive disease and survival is an area for further future investigation in a larger study population.

The recognized dose‐limiting toxicities for vinblastine and toceranib phosphate are neutropenia and gastrointestinal disease, respectively. The toxicity profile of these drugs in combination was established by Robat *et al*. (2012). Adverse effects observed in this study population were analogous to the previously published phase I study, however, differed in magnitude and severity (Robat *et al*. 2012). Sixty‐eight per cent (*n* = 27/40) of patients in this study experienced adverse effects. Comparatively, vinblastine and prednisolone chemotherapy have demonstrated a rate of toxicity of 6–29% (Thamm *et al*. 2006; Hayes *et al*. 2007; Serra Varela *et al*. 2016). No novel adverse effects were seen in this cohort.

Myelosuppression had lower frequency and severity in our cohort than previously reported by Robat *et al*. (2012). It should be considered though, that CBC was performed on a fortnightly basis, while the nadir for myelosuppression for vinblastine is 4–9 days and recovery at 7–14 days post‐treatment (Plumb, 2011). This will have caused an underestimation of myelosuppression in these patients. Neutropenia was seen ranging in severity from grade I‐III. Previous data noted several animals with grade III‐IV neutropenia (Robat *et al*. 2012). Adverse effects observed in this study were predominantly related to the gastrointestinal and musculoskeletal systems. Similar to the study by Robat *et al*. (2012), most patients’ gastrointestinal effects were considered mild to moderate. Many of these patients experienced resolution of clinical signs with temporary discontinuation of toceranib phosphate mid‐protocol. The authors speculate that concurrent MCT paraneoplastic gastrointestinal effects may intensify medication side effects which are gastrointestinal in origin. Careful consideration of patient and client factors should be employed during case selection, as adverse effects, while generally mild to moderate, are more common with this protocol than with vinblastine and prednisolone.

The major limitations identified in this study include the small sample size available, contributing to type II error, which likely accounts for the lack of discernible statistical significance in the results, lack of a control group, as well as the retrospective nature of this study. Due to being performed retrospectively, there are a variety of different pathologists and referring veterinarians involved in many of these cases. This may have led to inconsistencies in histologic grading and, in the case of group 2, likely variability in surgical skill and technique when MCT resection was performed. Additionally, retrospective studies rely on the accuracy and completeness of medical records. The use of historical comparisons for assessing survival advantages is suboptimal. In order to further assess the efficacy of this relatively new chemotherapy protocol in the management of canine MCTs, prospective studies are required. Additionally, no molecular methods, such as c‐kit mutation testing, were performed on MCTs in this study due to lack of availability in Australia at the time the study was performed. Several molecular diagnostic methods have been developed and investigated, including c‐kit protein expression, Ki67, PCNA and AgNORs. Supplementation with molecular methods has been suggested to better predict outcomes and biological behaviour in MCTs (London *et al*. 1996; Bergman *et al*. 2004; Takeuchi *et al*. 2013; Vascellari *et al*. 2013; Patruno *et al*. 2014). The presence of c‐kit mutation, while indicating high biological aggressiveness, is thought to facilitate the effects of RTK‐inhibitor drugs on neoplastic cells (London *et al*. 2009; Yamada *et al*. 2011; Blackwood *et al*. 2012; Warland *et al*. 2015).

In conclusion, this study has introduced combination vinblastine and toceranib phosphate chemotherapy as a valuable and efficacious contribution to the therapeutic modalities available for the treatment of canine MCTs, and further reinforces the value of RTK inhibitors as a therapeutic option for canine MCTs. Additional prospective studies are required to more thoroughly characterize this protocol's role in various clinical settings in a larger patient population.

## Source of funding

This research received no specific funding support.

## Conflict of Interest

The authors do not disclose any conflicts of interest.

## Ethical statement

The authors confirm that the ethical policies of the journal, as noted on the journal's author guidelines page, have been adhered to. No ethical approval was required as this is a retrospective analysis, relying on medical records only.

## Contributions

MT and KO examined, treated and authored the medical records for all patients included in this study. JAO collated and performed statistical analysis of the data. JAO wrote the manuscript, with all authors reviewing and approving of the final paper.
